# Introducing Porphyrin Units by Random Copolymerization Into NDI-Based Acceptor for All Polymer Solar Cells

**DOI:** 10.3389/fchem.2020.00310

**Published:** 2020-04-28

**Authors:** Jinliang Liu, Mengzhen Li, Dong Chen, Bin Huang, Qiannan He, Shanshan Ding, Wenquan Xie, Feiyan Wu, Lie Chen, Yiwang Chen

**Affiliations:** ^1^College of Chemistry, Institute of Polymers and Energy Chemistry (IPEC), Nanchang University, Nanchang, China; ^2^Institute of Advanced Scientific Research (IASR), Jiangxi Normal University, Nanchang, China

**Keywords:** all-polymer solar cells, porphyrin, naphthalene diimide, random copolymerization, device performance

## Abstract

Naphthalene diimide (NDI)-based polymer N2200 is a promising organic polymer acceptor for all-polymer solar cells (all-PSCs), but its inherent shortcomings like poor extinction coefficient and strong aggregation limit further performance optimization of all-PSCs. Here, a series of random copolymers, PNDI-Px, were designed and synthesized by introducing porphyrin unit into NDI-based polymer as acceptors for all-PSCs. These random copolymers show a higher absorption coefficient and raised the lowest unoccupied molecular orbital (LUMO) energy levels compared to N2200. The crystallinity can also be fine-tuned by regulation of the content of porphyrin unit. The random copolymers are matched with polymer donor PBDB-T for the application in all-polymer solar cells. The best power conversion efficiency (PCE) of these PNDI-Px-based devices is 5.93%, ascribed to the overall enhanced device parameters compared with the N2200-based device. These results indicate that introducing porphyrin unit into polymer is a useful way to fine-tune the photoelectric performance for efficient all-PSCs.

## Introduction

Organic solar cells (OSCs) have caused substantial research attributed to their remarkable features such as wide sources of materials, flexibility, light weight, solution processing, high-throughput preparation, and so on (Li, 2012; Søndergaard et al., 2012; Su et al., 2012; Cui, 2018). Very recently, the power conversion efficiencies (PCEs) of OSCs based on non-fullerene small-molecule acceptors have exceeded 16% (Cui et al., 2019; Fan et al., 2019; Jiang et al., 2019; Xiong et al., 2019; Xu et al., 2019; Yuan et al., 2019; Liu et al., 2020). Among various types of OSCs, all-polymer solar cells (all-PSCs) with active layer composed of a p-type polymer donor and an n-type polymer acceptor have attracted consistently increasing interest and research. The main reasons are their unparalleled advantages of optical and electronic properties, tunability, eminent mechanical flexibility, and excellent device stability (Diao et al., 2015; Kim et al., 2015; Kang et al., 2016; Liu et al., 2018; Zhang et al., 2018; Zhou and Facchetti, 2018), which is propitious to practical applications such as large-area flexible and stable devices. With the renewal of materials and the optimization of device processing technology, the PCE of all-PSCs have reached a value over 11% (Li et al., 2019a,b; Meng et al., 2019; Wu et al., 2019; Zhu et al., 2019). Even so, the efficiency of all-PSCs is still in arrears compared to the OSCs based on small molecule, due to the insufficient high-performance n-type polymer materials.

Generally, the high-quality n-type polymer materials are primarily based on the functionalized arenes of imides, e.g., diketopyrrolopyrrole (DPP) (Li et al., 2014, 2015; Zhang and Jin, 2019), naphthalene diimide (NDI) (Bhosale et al., 2008), perylene diimide (PDI) (Seo et al., 2011), bithiophene imide (BTI) (Wang et al., 2017), and double B←N (boron–nitrogen coordination bond) bridged bipyridine (BNBP) (Dou et al., 2016). In these various n-type organic semiconductors materials, NDI-based copolymers, especially poly(2,7-bis(2-octyldodecyl)benzo[lmn][3,8]phenanthroline-1,3,6,8(2H,7H)-tetraone-4,9-diyl)([2,2′]bithiophenyl-5,5′-diyl) (N2200), are the most outstanding one as all-PSC acceptor because of its good solubility and high electron mobility (Yan et al., 2009; Gao et al., 2016). Nevertheless, its inherent shortcomings such as poor extinction coefficient and strong aggregation limit the advancement of short circuit current (*J*_*SC*_) and fill factor (FF), further limiting the PCE of all-PSCs (Schubert et al., 2012; Kang et al., 2016). In recent years, many research groups have been successful in introducing the third unit to regulate the photovoltaic performance of polymer acceptors through random copolymerization (Li et al., 2016; Chen et al., 2018; Liu et al., 2018; Xu et al., 2018; Kolhe et al., 2019). For most random copolymer-based systems, the morphology of the active layer can be fine-tuned through regulation of the crystallinity of the random copolymers.

Apart from optimizing the morphology of the bulk-heterojunction, the strong and broadened light absorption of polymer acceptor is also critical to optimize the performance of all-PSCs. Recently, our group introduced the dye group, 2-(1, 1′-dicyanomethylene)-4-(3-thienylmethylene) rhodanine (TR), into the NDI-based acceptor, obtaining improved extinction coefficient, raised the lowest unoccupied molecular orbital (LUMO) energy levels, and reduced crystallization (Chen et al., 2018). Inspired by this strategy, we are interested in introducing other chromophores into terpolymers to improve the absorption of polymers. Porphyrin as a type of chromophore has many remarkable features, like excellent light harvesters, easily fine-tuning optical and electronic properties and efficient electron transfer (Lee et al., 2018; Mahmood et al., 2018), which is in favor of polymer material performance improvement. Li and coworkers successfully combined porphyrin group with perylene bisimides to obtain efficient small-molecule acceptors (SMAs) with high extinction coefficient (over 2.0 × 10^5^ cm^−1^) for OSCs (Zhang et al., 2017; Guo et al., 2018). These results motivated us to introduce porphyrin units into polymer acceptor which is rarely reported.

Herein, we introduced 5,15-dibromo-10,20-bis(4-octylphenyl) porphyrin Zinc block as the third unit into N2200 to partly replace the 2,2-bithiophene blocks by random polymerization, obtaining a series of random copolymer PNDI-Px [x represents the percentage of porphyrin (P) fusion ring relative to total acceptor units]. We systematically studied the absorption, energy levels, and crystallinity of polymer PNDI-Px. Ma and co-workers have previously reported that PBDB-T:N2200 has high tolerance to blend ratios (Zhang et al., 2018), and in view of the principle of complementary absorption and energy level matching, solar cells were prepared utilizing PBDB-T as donor and PNDI-Px as acceptor in the active layer. We compared the light harvesting, film morphology, exciton dissociation, charge transport, and the resulting photovoltaic performance of PBDB-T:PNDI-Px and PBDB-T:N2200 blends. With appropriate loadings of the porphyrin unit, the random copolymers have stronger absorption coefficient, up-shifted LUMO energy levels, more flexible main chain, and lower aggregation. Eventually, the device constructed from a PBDB-T:PNDI-P10 blend film generated an improved PCE (5.93%), with overall improved device parameters including open-circuit voltage (*V*_OC_) = 0.86 V, *J*_SC_ = 12.84 mA/cm^2^, and *FF* = 54.34%, compared with N2200-based device.

## Results and Discussion

### Material Synthesis and Characterization

**Scheme 1:**
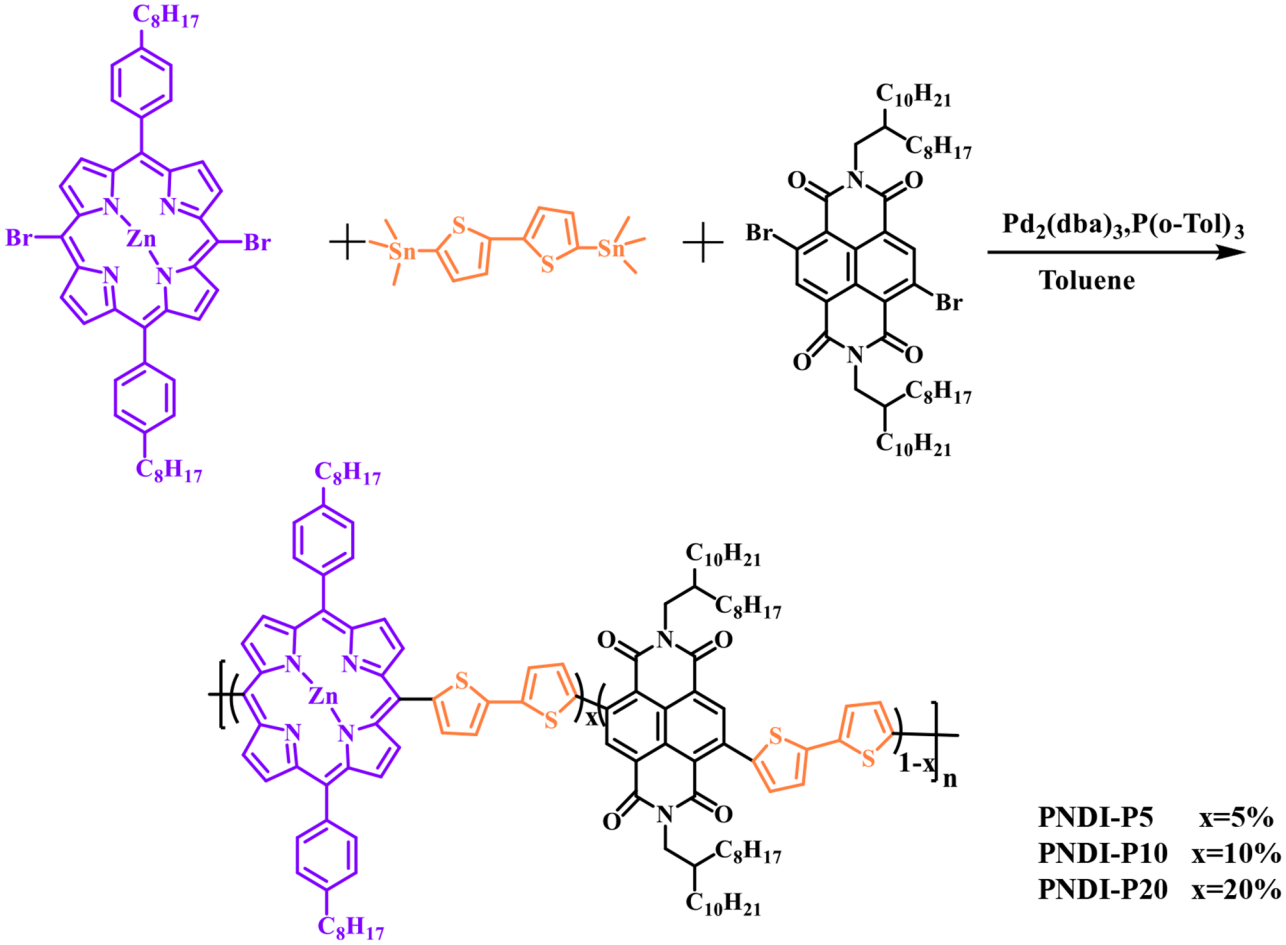
Structure and synthesis routes of PNDI-Px.

The new random copolymers PNDI-Px were synthesized by stille coupling polymerization of three monomers, 4,9-Dibromo-2,7-bis(2-octyldodecyl)benzo[lmn][3,8]phenanthroline-1,3,6,8(2H,7H)-tetraone (NDIBr_2_), 5,15-dibromo-10,20-bis(4-octylphenyl)porphyrin zinc, and 5,5′-bis(trimethylstannyl)-2,2′-bithiophene (Scheme 1). The detailed procedures are summarized in the supporting information. All the novel random polymers dissolve well in common organic solvents like tetrahydrofuran (THF), chlorobenzene (CB), o-dichlorobenzene (o-DCB) as well as chloroform (CF). The NMR spectra were used to confirm the structures of the copolymers (Figures S1–S3, Supporting Information). Thermogravimetric analysis (TGA) shows that the thermal decomposition temperature (T_d_) of N2200 and PNDI-Px is about 420°C at a weight loss of 5% (Figure S4), which indicates that the polymers have sufficient thermal stability in future electronic device applications. The number average molecular weights (Mn) of the polymers were measured by the Waters gel permeation chromatography with THF as solvent, as shown in Figure S5 and Table 1. The Mn values of PNDI-P5, PNDI-P10, and PNDI-P20 are 265.4, 250.4, and 284.7 kDa with the corresponding polydispersity index (PDI) of 2.12, 2.32, and 2.29, respectively.

**Table 1:** Molecular weight, thermal transition, and optical properties of polymer PNDI-P(x) and N2200.

**Polymer**	**Mn (kDa)**	**PDI**	***T*_**m**_ (^**°**^C)**	***T*_**c**_ (^**°**^C)**	**λ_max_ (nm)**	***E*$E_{\text{g}}^{\text{opt}}$ (eV)**
N2200	_	_	330.04	298.46	706	1.50
PNDI-P5	265.4	2.12	319.25	293.11	707	1.46
PNDI-P10	250.4	2.32	320.78	291.58	695	1.47
PNDI-P20	284.7	2.29	345.40	314.25	683	1.47

**Figure 1:**
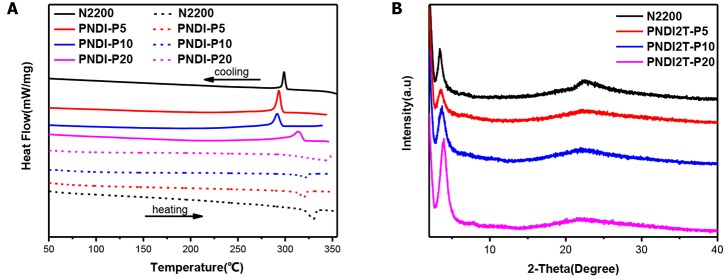
**(A)** DSC thermograms of neat PNDI-Px and N2200, measured with a scan rate of 10°C per minute. **(B)** X-ray diffraction patterns of the polymer PNDI-Px and N2200 in film.

The solid-state transition state of the polymers was characterized by differential scanning calorimetry (DSC) and depicted in Figure 1A and Table 1. All the new acceptor copolymers show only one melting transition on heating and one crystallization transition on cooling processing, confirming that these copolymers are random copolymers but not the block copolymers or the mixture of two alternative copolymers. N2200 has a melting temperature (T_m_) of up to 330°C, which is relatively high in conjugated polymers. The new random copolymers of PNDI-P5 and PNDI-P10 clearly present the reduced Tm and crystallization temperature (T_c_). It is because introducing third units into the polymer backbone by random copolymerization often reduces the ordering of the crystallinity of resulting copolymers (Li et al., 2016). However, for PNDI-P20, too many porphyrin units included in the backbone results in the slightly improved *T*_m_ and *T*_c_, probably caused by the stronger intramolecular interactions from the large conjugate plane of porphyrin. So as to deeply study the molecular packing and crystallization of new random copolymer PNDI-Px, X-ray diffraction (XRD) was employed (Figure 1B). It can be clearly seen from the XRD plot that as the amount of porphyrin units increases, the diffraction peak of the PNDI-Px film decreases first and then increases at low angles, implying that crystallinity of the PNDI-Px also decreases first and then increases. It reveals that random copolymerization of small loadings of the porphyrin group into the backbone can reduce regularity of molecular chains and obtain more flexible backbone, which facilitate the interface contact between donor and acceptor and, consequently, optimization of morphology, whereas the loading of excessive porphyrin leads to greater crystallization, probably due to the large conjugate plane of the porphyrin itself. This phenomenon is in accordance with the DSC observation.

**Figure 2:**
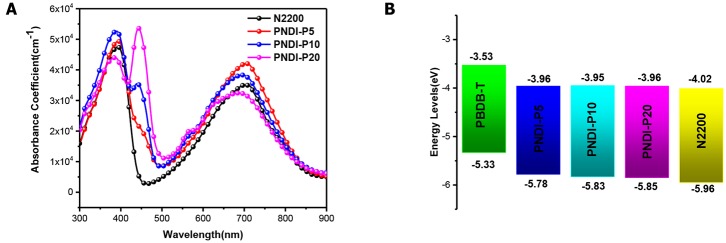
**(A)** Absorption coefficients of polymer PNDI-Px and N2200 in thin film. **(B)** Energy levels of PBDB-T, PNDI-Px and N2200.

The UV-Vis absorption spectra described the optical absorption properties of N2200 and PNDI-Px in CF solutions and thin films (Figure 2A, Figure S6). In solution and film, the absorption curves of PNDI-P5, PNDI-P10, and PNDI-P20 are similar to N2200, with two different absorption bands centered at 320–420 and 570–780 nm, which is caused by the excitations with the π-π^*^ manifolds of local NDI and intramolecular charge transfer (ICT) character, respectively. Strikingly, as the content of porphyrin incorporation increases, the absorption peak (absorption bands centered at 420–470 nm) of porphyrin fusion ring becomes more and more obvious. The absorption intensity of PNDI-P20 at 420–470 nm is most pronounced, proving the highest loading of porphyrin. In addition, the absorption intensity at 570–780 nm of copolymer PNDI-P5 and PNDI-P10 is also enhanced compared with the N2200. Therefore, incorporation of the third porphyrin unit by random copolymerization is a useful way to increase the light-harvesting of the NDI-based polymer acceptor. Compared with N2200, the absorption intensity of PNDI-P20 at 570–780 nm is slightly decreased, which is mainly due to the increase of molecular disorder and decrease of intramolecular interaction caused by the introduction of porphyrin. As shown in Figure 2A, with the increase of porphyrin content, the absorption coefficient of PNDI-Px film gradually improved in comparison to N2200. The absorption coefficients for N2200, PNDI-P5, PNDI-P10, and PNDI-P20 film are 4.7 × 10^4^, 5.0 × 10^4^, 5.3 × 10^4^, and 5.4 × 10^4^ cm^−1^, respectively. Similarly, the absorption coefficients for N2200, PNDI-P5, PNDI-P10, and PNDI-P20 solution are 28.8, 35.1, 35.6, and 38.1 L g^−1^·cm^−1^, respectively, showing the same tendency to those of films (Figure S6). In the light of the film absorption onsets, the optical bandgaps (Eg) of N2200, PNDI-P5, PNDI-P10, and PNDI-P20 are estimated to be 1.44, 1.53, 1.55, and 1.56 eV, respectively (Table 1).

The LUMO and highest occupied molecular orbital (HOMO) energy levels of PNDI-Px, N2200, and ferrocene were evaluated by cyclic voltammetry (CV). As depicted in the energy level diagrams (Figure 2B, Figure S7), the values of LUMO energy level for N2200, PNDI-P5, PNDI-P10, and PNDI-P20 are −4.01, −3.96, −3.95, and −3.96 eV, respectively. The values of HOMO energy level for N2200, PNDI-P5, PNDI-P10, and PNDI-P20 are −5.95, −5.78, −5.83, and −5.85 eV, respectively, showing that introduction of porphyrin units into the NDI-based copolymer can effectively raise LUMO and HOMO energy levels. The raised LUMO energy levels would be conducive to realize high *V*_OC_ in the OSC device.

### Photovoltaic Properties

**Figure 3:**
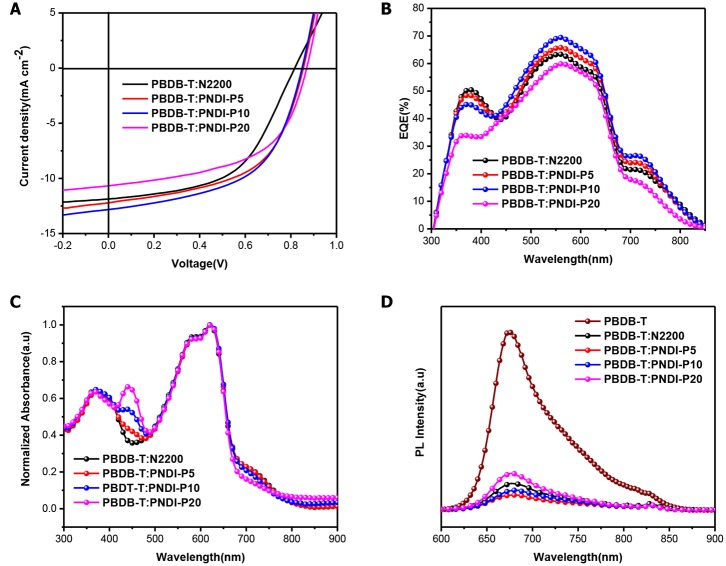
**(A)** J–V curves of PBDB-T:PNDI-P(x) (2:1 w:w) and PBDB-T:N2200 (2:1 w:w) solar cells. **(B)** EQE spectrograms for the optimal PBDB-T: PNDI-Px all-polymer solar cells. **(C)** Normalized optical absorption of PBDB-T:PNDI-P(x) (2:1 w:w) and PBDBT:N2200 (2:1 w:w) blend films. **(D)** PL emission spectra (580 nm excitation) of neat PBDB-T donor film and PBDB-T:PNDI-P(x) blend films.

All-PSCs is prepared to characterize the photovoltaic properties of novel terpolymer acceptors. The photovoltaic devices based on PBDB-T:PNDI-Px and PBDB-T:N2200 have the conventional construction of indium tin oxide (ITO)/ZnO/PBDB-T:acceptor/MoO_3_/Ag fabricated with CB as the processing solvent. The active layer of PBDB-T:PNDI-Px and PBDB-T:N2200 was fabricated with the ratio 2:1 (wt:wt) and annealed at 150°C for 10 min without using any solvent additives or other special treatments. The current density–voltage (J–V) curves of the all-PSCs are depicted in Figure 3A and the corresponding parameters are presented in Table 2. The PBDB-T:N2200 solar cell exhibits a PCE of 5.27% with a *V*_OC_ of 0.82 V, a *J*_SC_ of 11.87 mA/cm^2^, and a *FF* of 54.16%, which is well-consistent with the devices we reported previously (Chen et al., 2018). The PBDB-T:PNDI-P5 solar cell presents overall improved device parameters in comparison to PBDB-T:N2200 cells, including a superior PCE of 5.86%, a higher *J*_*SC*_ of 12.21 mA/cm^2^, a higher *V*_OC_ of 0.86, and a higher *FF* of 55.99%. The overall enhanced device parameters of *J*_SC_, *V*_OC_, and *FF* can be attributed to improved extinction coefficient, raised LUMO level and reduced crystallization, respectively. Furthermore, the device performance based on PNDI-P10 is further improved and the best device is obtained, which is mainly due to the further improvement of the absorption coefficient of PNDI-P10. The champion device with the PBDB-T:PNDI-P10 active layer shows a PCE of 5.93% with a *J*_SC_ of 12.84 mA/cm^2^, a *V*_OC_ of 0.85 V, and a *FF* of 54.34%. Besides, the PNDI-P20-based device also exhibits a higher *V*_OC_ and *FF*, but *J*_SC_ dropped significantly, which is ascribed to the increased crystallinity of PBDB-T:PNDI-P20 blended films.

**Table 2:** Photovoltaic properties of thermally annealed (150°C for 10 min) PBDB-T: N2200 (2:1 wt/wt) and PBDB-T: PNDI-P(x) (2:1 wt/wt) all-polymer solar cells.

**Active layer**	***V*_**OC**_ [V]**	***J*_**SC**_ [mA cm^**−1**^]**	**FF [%]**	**PCE [%]**
PBDB-T: N2200	0.82	11.87 [11.32]^a^	54.16	5.27 [5.15]^b^
PBDB-T: PNDI-P5	0.86	12.21 [11.79]	55.99	5.86 [5.77]
PBDB-T: PNDI-P10	0.85	12.84 [12.32]	54.34	5.93 [5.86]
PBDB-T: PNDI-P20	0.87	10.67 [10.09]	54.31	5.04 [4.92]

a *The J_SC_ integrated from the EQE spectrum*.

b *The values in the square brackets stand for the average PCEs from the 10 devices*.

The external quantum efficiency (EQE) spectrograms of the all-PSCs are plotted in Figure 3B. Similar to the blend film UV results (Figure 3C), the EQE curves of the PBDB-T:PNDI-Px-based cells exhibit response over the region between 300 and 850 nm. The PBDB-T:PNDI-P5-based and the PBDB-T:PNDI-P10-based devices show significant enhancement over the entire spectral response range relative to PBDB-T:N2200 cells, while the PBDB-T:PNDI-P20-based cell presents the weaker EQE response. As shown in Table 2, the integral *J*_SC_ values of EQE curve are in keeping with the values from J–V curves with mismatch <6%.

The photoluminescence (PL) quenching of the blend film was tested by 580 nm excitation light to quantitatively investigate the degree of exciton dissociation in the active layer. As dropped in Figure 3D and integrated in Table S1, the PL quenching efficiency (ΔPL) of PBDB-T blends is 85% for N2200, 92% for PNDI-P5, 89% for PNDI-P10, and 79% for PNDI-P20. These results indicate that the porphyrin-introduced PNDI-Px-based random copolymer semiconductor can effectively promote exciton dissociation and charge transfer. Meanwhile, the lower ΔPL in the PBDB-T:PNDI-P20 blend illustrates inefficient exciton diffusion and dissociation, which can partly explain its highest light absorption but with the lowest *J*_SC_ value.

### Morphology Characterization

**Figure 4:**
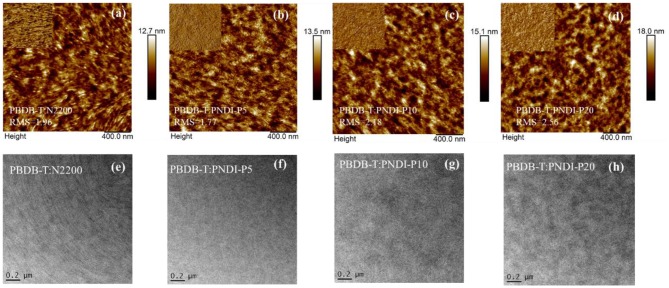
**(a–d)** AFM images of PBDB-T:PNDI-P(x) (2:1 w:w) and PBDBT:N2200 (2:1 w:w) blend films. **(e–h)** TEM images of PBDB-T:PNDI-P(x) (2:1 w:w) and PBDB-T:N2200 (2:1 w:w) blended films on the actual optimum devices.

To deeply understand the influence of the porphyrin-incorporated polymer acceptors on the morphology of bulk heterojunctions, the surface morphology of the PBDB-T:N2200 and PBDB-T:PNDI-Px blend films were investigated by atomic force microscopy (AFM) and transmission electron microscopy (TEM). As plotted in Figures 4a–d, all the blend films exhibit smooth surfaces with similar RMS roughness values between 1.77 and 2.56 nm. Among these blend films, PBDB-T:PNDI-P5 possesses the most uniform fibril nanophase separation with a suitable phase separation size, which explained its best *FF*. The PBDB-T:PNDI-P10 blended film shows fibril nanophase separation in comparison to PBDB-T:N2200 blended film, in favor of the exciton dissociation, charge transport, and collection. The morphology of PBDB-T:PNDI-P20 blended film presents a slightly bigger aggregation, leading to the reduced *FF* and *J*_SC_. As shown in Figures 4e–h, this morphological evolution can also be obviously detected by TEM observation. It can be deduced that a proper reduction in the crystallinity of N2200 can boost donor penetration of the acceptor, form an interpenetrating network structure, and have a more suitable domain size and phase separation.

## Conclusions

To sum up, we designed and synthesized a series of novel random copolymers of PNDI-Px that introduce porphyrin into the NDI-based acceptor by random polymerization. Compared with N2200, the new polymer acceptors yield significantly higher absorbance coefficient, up-lying LUMO energy levels, lower crystallinity, and improved film morphology, accompanied by higher *J*_SC_ and *V*_OC_ values in solar cells. The polymer PNDI-P10 matched with the donor PBDB-T exhibits champion performance with a PCE of 5.93% with a *J*_SC_ of 12.84 mA/cm^2^, a *V*_OC_ of 0.85 V, and a *FF* of 54.34%, which are higher than the N2200-based device. These results demonstrate that the incorporation of the third component porphyrin with excellent light harvester ability in the polymer acceptor has great potential in adjusting light absorption coefficient, crystallinity, and phase separation size of all-PSCs.

## Data Availability Statement

All datasets generated for this study are included in the article and/or the Supplementary Material.

## Author Contributions

JL and DC designed, synthesized, and characterized polymeric acceptors. ML, BH, and QH fabricated and characterized all-PSCs devices. SD and WX characterized the morphology of active layer. LC, FW, and YC guided material synthesis, device preparation, and characterization. JL and LC wrote the manuscript. All authors were responsible for discussing the results.

## Conflict of Interest

The authors declare that the research was conducted in the absence of any commercial or financial relationships that could be construed as a potential conflict of interest.
