# Supplementary material for: Introducing Porphyrin Units by Random Copolymerization Into NDI-Based Acceptor for All Polymer Solar Cells
Source: Front Chem. 2020 Apr 28;8:310. doi: 10.3389/fchem.2020.00310 (PMC7199102; doi:10.3389/fchem.2020.00310)
Supplement: Supplementary file 1 [file Table_1.DOCX]

**Introducing Porphyrin Units by** **Random Copolymerization into NDI-based Acceptor for All Polymer Solar Cells**

Jinliang Liu^1^, Mengzhen Li^1^, Dong Chen^1^, Bin Huang^1^, Qiannan He^1^, Shanshan Ding^1^, Wenquan Xie^1^, Feiyan Wu*^1^, Lie Chen*^1^, Yiwang Chen^1,2^

^1^College of Chemistry/Institute of Polymers and Energy Chemistry (IPEC), Nanchang University, 999 Xuefu Avenue Nanchang 330031, (P. R. China).

^2^Institute of Advanced Scientific Research (IASR), Jiangxi Normal University, 99 Ziyang Avenue, Nanchang 330022, China.

*Corresponding author. Tel.: +86 791 8396830; fax: +86 791 83969830. E-mail: feiywu@ncu.edu.cn (F. Wu), chenlie@ncu.edu.cn (L. Chen).

**Keywords:** All-polymer solar cells; Porphyrin; Naphthalene diimide; Random copolymerization; Device performance

**Experimental Section**

**Materials and Characterization**

The synthetic procedures were performed under argon atmosphere. Commercial chemicals (from Alfa, J&K Chemical, Energy Chemical, and Derthon) were used as received. 5,15-dibromo-10,20-bis-((4-octyl)-phenyl)-porphyrin zinc (II) were prepared according to literature procedures (Gao et al., 2015; Huang et al., 2012).

**Synthesis of Polymer PNDI-P5**:

2,6-Dibromonaphthalene-1,4,5,8-tetracarboxylic-N,N′-bis(2-octyldodecyl)diimide (NDIBr_2_) (0.19 mmol, 187.16 mg), 5,5′-bis(trimethylstannyl)-2,2′-bithiophene (2T) (0.2 mmol, 98.37 mg), 5,15-dibromo-10,20-bis-((4-octyl)-phenyl)-porphyrin zinc (II) (0.01 mmol, 9.08mg), tris(dibenzylideneacetone)dipalladium(0)(Pd2(dba)3) (3.66 mg) and tri(o-tolyl) phosphine (P(o-Tol)3) (4.86 mg) were dissolved in anhydrous toluene (10 mL) under a nitrogen atmosphere. The reaction mixture was heated at 100 °C with vigorous stirring for 48 h. After cooling to room temperature, the polymer was precipitated from the solution into acetone and was collected by filtration through a 0.45 μm Teflon filter. Then the polymer was washed in a Soxhlet extractor with acetone, hexane and chloroform. The chloroform fraction was purified by passing it through a short silica gel column and then precipitated from acetone again. Finally, the polymer was obtained by filtration through 0.45 μm Teflon filter and dried under vacuum at 40 °C overnight. Yield PNDI-P5 as a dark blue solid 135 mg (78%). ^1^H NMR (400 MHz, cdcl3) δ 8.82 (s, 1H), 8.50 (s, 2H), 7.52 (s, 1H), 6.97 (s, 2H), 4.15 (s, 1H), 3.49 (s, 4H), 2.27 (s, 3H), 1.86 (s, 11H), 1.81 – 0.39 (m, 387H), 0.21 (s, 4H), 0.09 (d, J = 19.8 Hz, 177H), -0.09 (s, 1H), -0.16 (s, 2H).

**Synthesis of Polymer PNDI-P10**:

It was prepared by the same procedure as described above, starting with NDI (0.16 mmol, 157.6mg), 2T (0.2 mmol, 98.37 mg), and 5,15-dibromo-10,20-bis-((4-octyl)-phenyl)-porphyrin zinc (II) (0.04 mmol, 36.32 mg). Yield: 126 mg (80%). ^1^H NMR (400 MHz, cdcl3) δ 8.83 (s, 1H), 8.52 (s, 3H), 7.52 (s, 1H), 6.99 (s, 2H), 5.12 (s, 1H), 4.11 (s, 1H), 3.52 (s, 1H), 2.25 (s, 4H), 2.00 (s, 9H), 1.21 (d, J = 280.3 Hz, 294H), 0.23 (s, 3H), 0.15 – -0.10 (m, 148H), -0.16 (s, 2H).

**Synthesis of Polymer PNDI-P20**:

It was prepared by the same procedure as described above, starting with NDI (0.18 mmol, 177.31 mg), 2T (0.2 mmol, 98.37 mg), and 5,15-dibromo-10,20-bis-((4-octyl)-phenyl)-porphyrin zinc (II) (0.02 mmol, 36.32 mg). Yield: 126 mg (76%). ^1^H NMR (400 MHz, cdcl3) δ 8.83 (s, 1H), 8.49 (s, 1H), 7.52 (s, 2H), 7.02 (s, 2H), 4.14 (s, 1H), 3.87 (s, 1H), 3.53 (s, 2H), 2.24 (s, 3H), 2.05 (s, 8H), 1.84 – 0.37 (m, 286H), 0.22 (s, 2H), 0.03 (d, J = 28.0 Hz, 146H), -0.10 (s, 3H).

**General Measurement**

The ^1^H spectra were recorded in deuterated solvents on a Bruker ADVANCE 400 NMR Spectrometer. ^1^H NMR chemical shifts were reported in ppm downfield from tetramethylsilane (TMS) reference using the residual protonated solvent as an internal standard. Thermogravimetric analysis (TGA) was carried out on a PerkinElmer TGA S-27 instrument for thermal analysis at a heating rate of 10 °C/min under nitrogen. Number-average (Mn) and weight-average (Mw) molecular weights were determined with Waters 2414 gel permeation chromatography (GPC) with a refractive index detector in THF using a calibration of polystyrene standards. X-ray diffraction (XRD) measurements were performed with a igaku D/Max-B X-ray diffractometer with Bragg-Brentano parafocusing geometry, a diffracted beam monochromator, and a conventional cobalt target X-ray tube set to 40 KV and 30 Ma.

1. **Electrochemical characterizations**

Cyclic voltammetry was performed under an inert atmosphere at a scan rate of 0.1 V s^-1^ and 1 M tetrabutylammonium hexafluorophosphate in acetonitrile as the electrolyte, a glassy-carbon working electrode coated with samples, a platinum-wire auxiliary electrode, and an Ag/AgCl as a reference electrode.

1. **Optical characterizations**

UV-vis absorption spectra were recorded on a Perkin Elmer Lambda 750 spectrophotometer. All film samples were spin-cast on quartz slice substrates. Solution UV-Vis absorption spectra also were collected on a Perkin Elmer Lambda 750 Spectrophotometer. A cuvette with a stopper (Sigma Z600628) was used to avoid volatilization during the measurement.

1. **Device Fabrication and Characterizations**.

All the devices were manufactured with the structure of Glass/ITO/ ZnO /active layer/MoO3/Ag. The conductive ITO substrates were sequentially cleaned with ultrasonication in acetone, detergent, water, and isopropanol. After drying the ITO substrates and treating the surface with UV ozone for 20 min, The ZnO precursor solution was prepared by dissolving 0.5 g of zinc acetate dihydrate (Zn(CH3COO)2·2H2O, 99.9%, Alfa) and 138 ul of ethanolamine (NH2CH2CH2OH, 99.5%, J&K) in 5 ml of 2-methoxyethanol (CH3OCH2CH2OH, 99.8%, Alfa). The ZnO precursor was spin-coated at 4000 r.p.m. for 1 min onto the ITO surface. After being baked at 200 °C for 60 min in air, the ZnO-coated substrates were transferred into a nitrogen-filled glove box. Then the active layer was prepared by spin-casting chlorobenzene solution with a concentration of 15 mg mL^-1^ in chlorobenzene solution to obtain about 100 nm and then annealed at 150 °C for 10 min blends film in a nitrogen-filled glove box. Subsequently, the double-layer structure of MoO3 (7 nm)/Ag (90 nm) was deposited over the active layer by thermal evaporation under a vacuum chamber to accomplish the device fabrication. The effective area of one cell was 0.04 cm^2^. The current−voltage (J−V) characteristics were measured by a Keithley 2400 Source Meter under simulated solar light (100 mW/cm^2^, AM 1.5 G, Abet Solar Simulator Sun2000). The incident photon-to-electron conversion efficiency (IPCE) spectra were detected on an IPCE measuring system (Oriel Cornerstone 260 1/4 m monochromator equipped with Oriel 70613NS QTH lamp). All the measurements were performed at room temperature under ambient atmosphere.

1. **Photoluminescence (PL) quenching characterization**

The photoluminescence (PL) spectra were measured by photoluminescence spectroscopy (Hitachi F-7000). The steady-state PL quenching efficiency (ΔPL) was estimated by the PL intensity of the PBDB-T: acceptor blends relative to that of the neat polymers. And ΔPL can be derived by using the following equation (Li et al., 2016).

$$\Delta PL=1-\frac{{PL}_{blend}}{{PL}_{polymer}}$$

1. **AFM and TEM characterizations.**

The specimen for MultiMode 8-HR (Bruker) atomic force microscopy (AFM) measurements was prepared using the same procedures those for fabricating devices but without MoO_3_/Ag on top of the active layer. Transmission electron microscopy (TEM) images were taken on a JEOL-2100F transmission electron microscope and an internal charge-coupled device (CCD) camera. The specimen for TEM measurement was prepared by spin casting the blend solution on ITO/PEDOT: PSS substrate, then floating the film on a water surface, and transferring to TEM grids.

**REFERENCES**

Chen, H. C., Lai, C. W., Wu, I. C., Pan, H. R., Chen, I. W. P., Peng, Y. K., et al. (2011). Enhanced performance and air stability of 3.2% hybrid solar cells: how the functional polymer and CdTe nanostructure boost the solar cell efficiency. *Adv. Mater.* 23, 5451-5455. doi: 10.1002/adma.201102775

Cheng, Y. J., Hsieh, C. H., Li, P. J., Hsu, C. S. (2011). Morphological stabilization by in situ polymerization of fullerene derivatives leading to efficient, thermally stable organic photovoltaics. Adv. Funct. Mater. 21, 1723-1732. doi: 10.1002/adfm.201002502

Gao, K., Li, L., Lai, T., Xiao, L., Huang, Y., Huang, F., et al. (2015). Deep absorbing porphyrin small molecule for high-performance organic solar cells with very low energy losses. *J. Am. Chem. Soc*. 137, 7282-7285. doi: 10.1021/jacs.5b03740

Huang, Y., Li, L., Peng, X., Peng, J., Cao, Y. (2012). Solution processed small molecule bulk heterojunction organic photovoltaics based on a conjugated donor–acceptor porphyrin. *J. Mater. Chem.* 22, 21841-21844. doi: 10.1039/C2JM34429G

Li, Z., Xu, X., Zhang, W., Meng, X., Ma, W., Yartsev, A., et al. (2016). High performance all-polymer solar cells by synergistic effects of fine-tuned crystallinity and solvent annealing. *J. Am. Chem. Soc*. 138, 10935-10944. doi:10.1021/jacs.6b04822

**Figure S1**. ^1^H NMR spectra of PNDI-P5 in CDCl3

**Figure S2**. ^1^H NMR spectra of PNDI-P10 in CDCl3

**Figure S3**. ^1^H NMR spectra of PNDI-P20 in CDCl3

**Figure S4.** Thermogravimetric analysis (TGA) plot of polymers PNDI-Px and N2200 with a heating rate of 10 ºC min^-1^ under nitrogen atmosphere.

**Figure S5**. GPC spectra of PNDI-Px in THF

**Figure S6.** a) Absorption coefficients of polymer PNDI-Px and N2200 in chloroform solutions, b)Normalized optical absorption of polymer N2200 and PNDI-Px in chloroform solutions, c) Normalized optical absorption of polymer N2200 and PNDI-Px in films.

**Figure S7**. Cyclic voltammetry (CV) of the PNDI-Px, N2200 and Ferrocene in the film measured in a 0.1 M Bu_4_NPF_6_-CH_3_CN solutions with a Pt electrode and an Ag/AgNO_3_ reference electrode.

**Table S1**. PL Quenching Efficiency of polymer and ITIC.

|  | PBDB-T: N2200 | PBDB-T: PNDI-P5 | PBDB-T: PNDI-P10 | PBDB-T: PNDI-P20 |
| --- | --- | --- | --- | --- |
| ΔPL | 85% | 92% | 89% | 79% |
